# 1184. Structural Characteristics of Alcohol Venues are Associated with Positive HIV Test Results in Rural KwaZulu-Natal, South Africa

**DOI:** 10.1093/ofid/ofac492.1019

**Published:** 2022-12-15

**Authors:** Sarah Norton, Neo Morojele, Gerald Friedland, Tony Moll, Sheela Shenoi

**Affiliations:** University of Utah, Salt Lake City, Utah; University of Johannesburg, Johannesburg, Gauteng, South Africa; Yale University School of Medicine, New Haven, Connecticut; Philanjalo NGO, Tugela Ferry, KwaZulu-Natal, South Africa; Yale University, New Haven, Connecticut

## Abstract

**Background:**

The HIV epidemic in South Africa is among the largest in the world with a prevalence of 20% among adults. The South African National Strategic Plan recognizes the need to address social and structural barriers to HIV prevention, as well as sociocultural and behavioral drivers, including alcohol abuse. Alcohol venues (AVs) are important social venues, yet few studies have explored structural factors contributing to HIV risk. We sought to evaluate the relationship between structural characteristics of AVs and HIV status of patrons.

**Methods:**

The study was conducted in rural Msinga, South Africa where, as part of a community-based approach, education, counseling and HIV testing was offered at a convenience sample of AVs. Nested within this study, staff completed a structural characteristic checklist of the AVs where testing occurred. Categorical analyses evaluated the association between structural characteristics and positive HIV results.

**Results:**

Of the 488 individuals tested at 46 AVs, 43 (8.8%) were seropositive. The majority of AVs were rural (71.7%), along a gravel road (43.5%), un-registered (56.5%), informal (73.9%), lacked a liquor license (58.7%), were well-maintained (78.3%), single rooms (84.1%) with lighting (97.8%), make-shift seating (62.2%), and adjacent to outdoor space (65.8%). Sound systems (41%) and bathrooms (37%) were less common. Preventative health signage for under-age drinking (26%), smoking cessation (8.7%) and HIV prevention (2.2%) were rare. Several AVs employed women to work behind the bar; security guards and DJs were less common. Higher HIV prevalence was significantly associated with AVs that were well-maintained (p=0.008), in town (p=0.006) compared to remote, had an indoor toilet (p=0.004), discrete gender bathrooms compared to a single bathroom (p=0.003), and a security guard present (p=0.047).

**Conclusion:**

Higher HIV prevalence was associated with certain structural characteristics of AVs. Further research is needed to understand social dynamics within AVs and how these structural characteristics facilitate risk behaviors to inform community-based interventions that can address HIV risk.

**Disclosures:**

**Sheela Shenoi, MD MPH**, Merck: My spouse worked for Merck 1997-2007 and retains stock in his retirement account. There is no conflict with the work presented in the abstract.

